# Supplementary material for: The Burden of Congenital Hypothyroidism Without Newborn Screening: Clinical and Cognitive Findings from a Multicenter Study in Algeria
Source: Int J Neonatal Screen. 2025 Sep 15;11(3):78. doi: 10.3390/ijns11030078 (PMC12452675; doi:10.3390/ijns11030078)
Supplement: Supplementary file 1 [file IJNS-11-00078-s001.zip › IJNS-3696432-supplementary.pdf]

**Age**		
Age at diagnosis	Mean ± SDS (Months)	8.6±20
	Median [range] (Months)	1.6 [0.05-150]
	Median [range] (Days)	48 [1.5-4500]
Age at first evaluation	Mean ± SDS (Months)	17.1±27.6
	Median [range] (Months)	4.5 [0.03-156]
Age at treatment	Mean ± SDS (Months)	10.2±21.4
	Median [range] (Months)	2 [0.06-150]
**Age distribution**		
< 1 Month	n,(%)	100(35%)
1-3 Months	n,(%)	81(28%)
3-6 Months	n,(%)	43(15%)
6-12 Months	n,(%)	12(4%)
>12 Months	n,(%)	52(18%)
**Sex Distribution**		
Female (F)	n,(%)	159(55%)
Male (M)	n,(%)	129(45%)
F to M ratio	Ratio	1.23
**Presenting problem leading to the diagnosis of CH**		
Prolonged neonatal jaundice	n,(%)	105(36.5%)
Constipation	n,(%)	43(15%)
Family screening	n,(%)	38(13%)
Psychomotor delay	n,(%)	41(14%)
Short Stature	n,(%)	22(8%)
Hypotonia	n,(%)	14(5%)
Macroglossia	n,(%)	6(2%)
Umbilical hernia	n,(%)	4(1%)
others	n,(%)	15(5%)
**Clinical signs at diagnosis**		
Prolonged jaundice	n,(%)	166(58%)
hypotonia	n,(%)	15(5%)
Macroglossia	n,(%)	20(7%)
Umbilical hernia	n,(%)	45(16%)
Disjunction of sutures	n,(%)	7(2%)
Hoarse voice	n,(%)	6(2%)
Prematurity (Gestational age< 37)	n,(%)	27(9%)
SGA*	n,(%)	33(11%)
Length at birth (LB)	Mean ± SDS (cm)	49.6±2.9
Weight at birth (WB)	Mean ± SDS (kg)	3.2±0.7
Head circumference at birth	Mean ± SDS (centiles)	34.4±2.3
Height at first presentation (SD)	Mean ± SDS (SD)	-1.5±1.9
Weight at first presentation (SD)	Mean ± SDS (SD)	-1.02±1.8
BMI at first presentation (SD)	Mean ± SDS (SD)	0.12±1.8

Table S1: Clinical characteristics at presentation

*SGA (Small for gestational age) if LB and/or WB were < -10th percentile

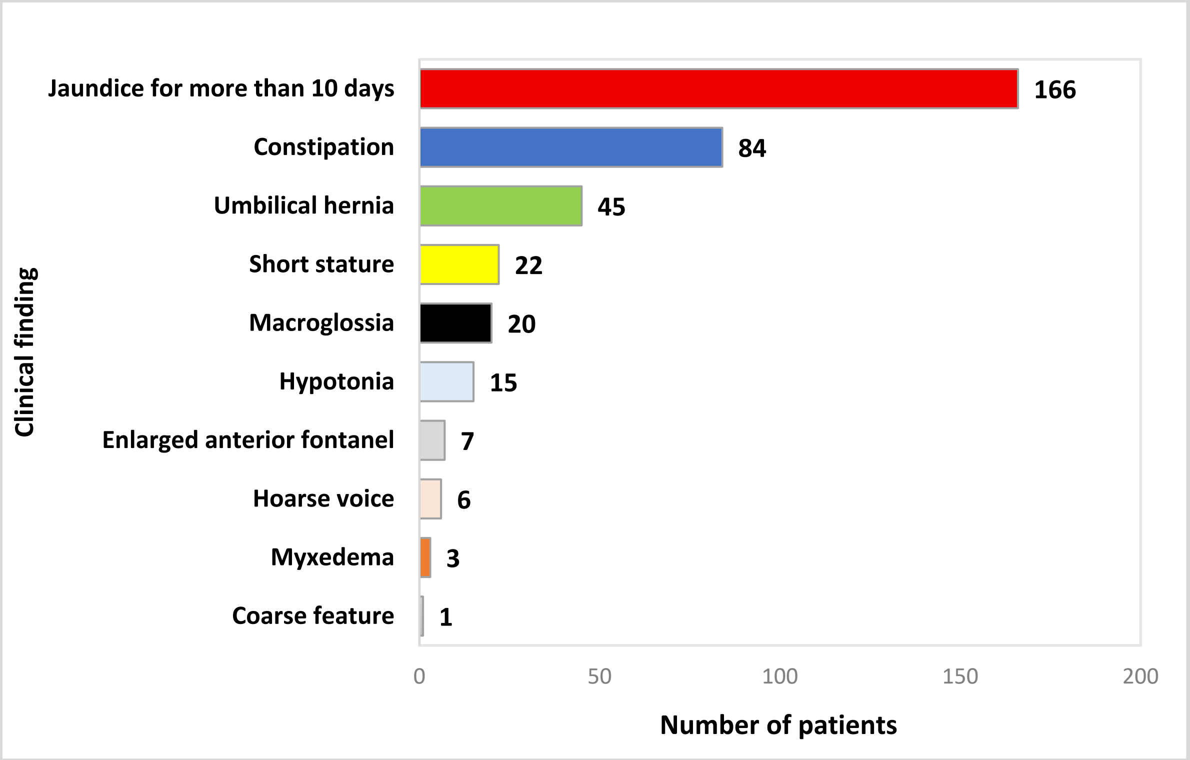

Figure S1: Clinical signs and symptoms of congenital hypothyroidism found at diagnosis

Table S2: Age at diagnosis according to the cause of referral

**Table S2: Age at diagnosis according to the cause of referral**			
Cause of referral/presenting problem	N	Age at diagnosis in Months (Mean± SD)	***p*<0.001**
Family Screening	38	1.02±2.04[0.07-12]	
Prolonged jaundice	100	1.66±3.54 [0.05-34]	
Hypotonia	9	5.18±5.72 [0.1-16]	
Constipation	18	8.05±11.05[0/26-43]	
Psychomotor delay	37	14.12±13.8[1-51]	**<0.0001
Short Stature	17	41.26±40.73[2.33-150]	***<0.0001

*ANOVA test comparing age at diagnosis according to the cause of referral

** Comparison between age at diagnosis in patients diagnosed with family screening and patients diagnosed with psychomotor delay

*** Comparison between age at diagnosis in patients diagnosed with family screening and patients diagnosed with short stature

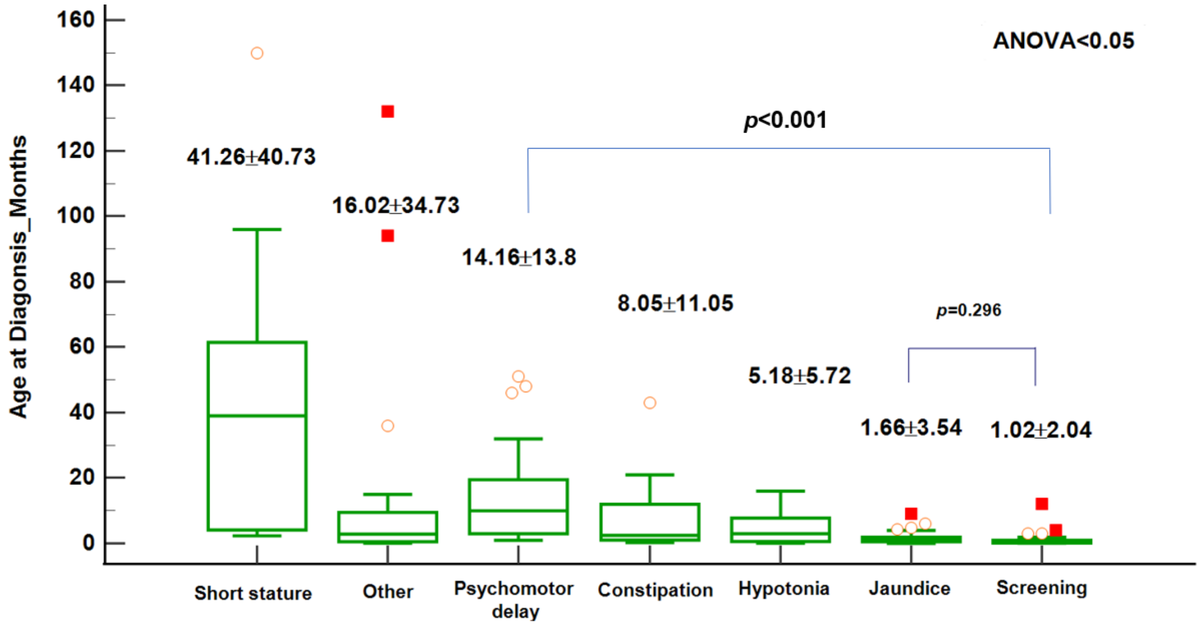

Figure S2: Age at diagnosis in months according to the cause of referral. NS: No significant difference.

Table S3: Assessment of the Initial treatment with Levothyroxine

**Age at start treatment**	**All patients**	**<1 Month**	**1-3 Months**	**3-12 Months**	**>12 Months**
**N,(%)**	288	82(28%)	89(31%)	54(19%)	62(22%)
**LTX Initial dose**<br>mean±SD [range] µg/kg/day	6.9±4.1<br>[0.6-25.6]	7.8±3.7<br>[1.1-18.4]	7.55±4.1<br>[1.2-20.3]	7.8±4.6<br>[0.8-25.6]	4.4±2.9<br>[0-13.3]
**Low dose**	149 (52%)	42(51%)	45(50.6%)	18(33%)	44(71%)
Dose of LTX< 9µg/kg/day (n,%)<br>Dose of LTX< 6µg/kg/day (n,%)		42(51%)<br>25(30%)	45(50.6%)<br>29(33%)	-<br>18(33%)	-<br>44(71%)
		***p*=0.001**			

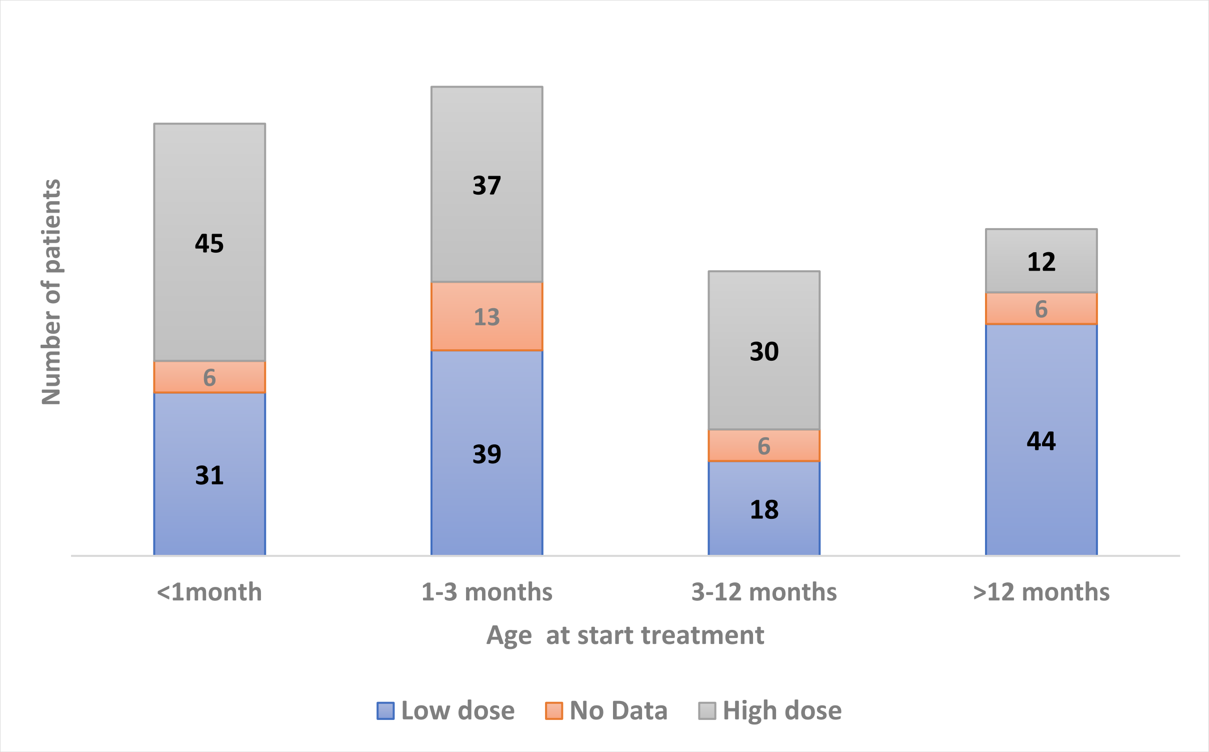

Figure S3: Dose L-T4 treatment at initiation by age

Table S4: Risk factor of Neurodevelopmental delay

Variable	*p*-value	ORs	95% CI
**Age > 2months**	**<0.0001**	**7.7220**	**2.8852 - 20.6673**
**Age<1month**	**0.0853**	**0.2945**	**0.0732 - 1.1854**
Umbilical hernia	0.0230	2.7986	1.1523 - 6.7972
Familial thyroid disease	0.0272	0.4415	0.2137 - 0.9121
Hypotonia	0.1331	2.6121	0.7462 - 9.1436
Related Malformation	0.0521	2.3631	0.9920 - 5.6291
Constipation	0.1955	1.5994	0.7855 - 3.2568
fT4<5pmol	0.1847	1.5893	0.8015 - 3.1513

Odds Ratios (ORs)
